# Pathogenic mechanism of extracranial arteriovenous malformations: insights from clinical, pathological, and genetic analyses

**DOI:** 10.1007/s00428-025-04158-7

**Published:** 2025-07-02

**Authors:** Katsutoshi Hirose, Yumiko Hori, Kazuaki Maruyama, Daisuke Motooka, Kenji Hata, Shinichiro Tahara, Takahiro Matsui, Satoshi Nojima, Masaharu Kohara, Kyoko Imanaka-Yoshida, Satoru Toyosawa, Eiichi Morii

**Affiliations:** 1 Department of Oral and Maxillofacial Pathology, The University of Osaka Graduate School of Dentistry, 1-8 Yamadaoka, Suita, Osaka 565-0871 Japan; 2 Department of Pathology, The University of Osaka Graduate School of Medicine, 2-2 Yamadaoka, Suita, Osaka 565-0871 Japan; 3 Department of Central Laboratory and Surgical Pathology, NHO Osaka National Hospital, 2-1-14 Hoenzaka, Chuo-Ku, Osaka 540-0006 Japan; 4 Department of Pathology and Matrix Biology, Mie University Graduate School of Medicine, 2-174 Edobashi, Tsu, Mie 514-8507 Japan; 5 NGS Core Facility, Research Institute for Microbial Diseases, The University of Osaka, 3-1 Yamadaoka, Suita, Osaka 565-0871 Japan; 6 Department of Molecular and Cellular Biochemistry, The University of Osaka Graduate School of Dentistry, 1-8 Yamadaoka, Suita, Osaka 565-0871 Japan; 7 Mie University Onco-Cardiology Research Center, 2-174 Edobashi, Tsu, Mie 514-8507 Japan

**Keywords:** MAP2K1, KRAS, BRAF, MAP4K4, Arteriovenous malformation, Spatial transcriptomics

## Abstract

Extracranial arteriovenous malformations (AVMs) are rare aggressive vascular malformations, and half of AVMs harbor mutations in the RAS/RAF/MAPK pathway. AVMs consist of abnormal networks of small vessels formed between arteries and veins. Although the abnormal small-vessel networks are considered to cause AVM progression, the underlying mechanisms remain poorly understood. This study elucidated the mechanisms underlying the pathogenesis of extracranial AVM in relation to genetic mutations. This retrospective clinical, pathological, and genetic study included 30 patients with extracranial AVMs. *MAP2K1*, *KRAS*, and *BRAF* mutations were identified in 14 (46.7%), 1 (3.3%), and 1 (3.3%) patient(s), respectively. Mutant AVMs were predominant in females, and histologically showed more infantile hemangioma-like small vessels and more enlarged small vessels inside the perineurium. Immunohistochemistry revealed high expression levels of phosphorylated ERK (extracellular signal regulated kinases), a downstream effector of the RAS/RAF/MAPK pathway, in AVMs regardless of mutational status or vessel type. Spatial transcriptomics revealed upregulation of genes involved in “positive regulation of cell adhesion”, “positive regulation of cell migration” and “blood vessel development” in the abnormal small vessels in *MAP2K1*-mutant AVMs. Among the up-regulated genes, MAP4K4, which regulates pathological angiogenesis and is a therapeutic target, was highly expressed in abnormal small vessels in AVMs. This study reports significant genotype–phenotype correlations in AVMs, indicating gene-specific effects. A detailed analysis of gene-specific effects, focusing especially on abnormal small-vessel networks, may offer insights into the mechanisms underlying AVM pathogenesis and its implications for targeted therapies.

**Supplementary Information:**

The online version contains supplementary material available at 10.1007/s00428-025-04158-7.

## Introduction

Vascular malformations are congenital vascular disorders that are classified according to their histological appearance [1]. Arteriovenous malformations (AVMs) are rare fast-flow vascular malformations consisting of abnormal networks of small vessels supplied by feeding arteries and draining into veins [2–4]. Most AVMs arise in the brain (brain AVMs), but AVMs can occur anywhere in the body (extracranial AVMs) [3, 4]. Extracranial AVMs occur most commonly in the head and neck regions, with a reported incidence of 2–7 persons per 1,000,000 population [2–5]. Extracranial AVMs are congenital, but symptoms typically emerge in adolescence or adulthood [2, 3, 6]. Extracranial AVMs are aggressive and can damage nearby structures with increased abnormal networking, leading to deformity, ischemia, pain, ulceration, bleeding, and cardiac failure [3, 6]. Extracranial AVMs progress throughout life, with treatment options limited and challenging, requiring embolization and/or surgical resection [3, 6, 7]. Complete surgical removal of the lesion is often not possible because extracranial AVMs are often diffuse and infiltrative, involving tissue planes in critical anatomic regions [3, 6]. Residual lesions commonly lead to AVM re-enlargement, with a recurrence developing during the first year [3, 6]. Most extracranial AVMs re-expand within 5 years [3, 6]. Although the abnormal networks of small vessels are fundamental to AVMs and are considered to cause AVM progression [4, 8], the underlying mechanisms remain poorly understood.

Recently, genetic studies have revealed that the mutations associated with a large proportion of vascular malformations are involved with the rat sarcoma virus gene (RAS)/rapidly accelerated fibrosarcoma gene (RAF)/mitogen-activated protein kinase (MAPK) pathway, or phosphoinositide 3-kinase (PI3K)/AKT pathway [1, 9]. The RAS/RAF/MAPK pathway is a critical signaling cascade of numerous cellular and developmental processes that regulate cell growth, differentiation, survival, and response to stress [10–12]. Oncogenic mutations in the RAS/RAF/MAPK pathway detected in various tumors have also been identified in extracranial/brain AVMs, suggesting that these mutations are involved in AVM pathogenesis [5, 13–16]. Approximately 50% of extracranial AVMs have a somatic gain-of-function mutation in *mitogen-activated protein kinase kinase 1* (*MAP2K1*) (Table 1) [5, 14–16]. Less frequently, gain-of-function mutations are observed in *Kirsten rat sarcoma viral oncogene* (*KRAS*) or *B-Raf proto-oncogene, serine/threonine kinase* (*BRAF*), which are upstream of *MAP2K1* in the RAS/RAF/MAPK pathway (Table 1) [5, 15, 16]. In vitro experiments using endothelial cells (ECs) of blood vessels derived from patients with *MAP2K1*- or *KRAS*-mutant AVMs show that the mutations occur within ECs but not in vascular smooth muscle or other stromal cells [14, 17–19]. Both mutations within ECs increase downstream effector, extracellular signal-regulated kinase (ERK) activity [14, 17–19]. Moreover, *MAP2K1*- or *KRAS*- mutant-transduced ECs enhance angiogenesis and migratory behavior, which may lead to abnormal coordination of artery-capillary-vein formation [15, 17, 19]. ECs-specific induced *MAP2K1*- or *KRAS*-mutations in zebrafish show abnormal networks connecting arteries and veins [15, 20]. Based on genetic data from previous studies, the RAS/RAF/MAPK pathway plays a central role in the pathogenesis of AVMs, and ECs harboring the mutations may act as key contributors to the formation of abnormal networks. In patients with AVM, the number of abnormal networks of small vessels increases gradually with worsening of the clinical condition [3, 6]. Therefore, a detailed analysis of these small-vessel networks, focusing especially on ECs, concerning genetic mutations may elucidate the mechanism underlying extracranial AVM progression.

**Table 1:** Summary of somatic mutations identified in the present study and other major genetic studies of extracranial arteriovenous malformations

Study	MAP2K1	KRAS	BRAF	RASA1	Mutation-negative	Screened cases (percent of mutant cases)
Couto et al. 2017.^14^	16	0	0	0	9	25 (64%)
Al-Olabi et al. 2018.^15^	4	3	1	0	15	23 (34.8%)
Sissy et al. 2022.^5^	7	6	2	2	6	23 (73.9%)
Present study	14	1	1	0	14	30 (53.3%)
Total (percent of mutation)	41 (40.6%)	10 (9.9%)	4 (4.0%)	2 (2.0%)	44 (43.6%)	101

*MAP2K1* mitogen-activated protein kinase kinase 1, *KRAS* KRAS proto-oncogene, GTPase, *BRAF* B-Raf proto-oncogene, serine/threonine kinase, *RASA1* RAS p21 protein activator 1

This study investigated the correlations between genetic mutational status and clinicopathological features to elucidate the mechanism underlying extracranial AVM pathogenesis in relation to genetic mutations. Furthermore, we examined the mRNA gene expression patterns in the ECs of *MAP2K1*-mutant AVMs using spatial transcriptomics to elucidate how endothelial *MAP2K1* mutation leads to AVM progression. To our knowledge, this is the largest series of genetically studied extracranial AVMs, and this is the first study to comprehensively examine the genetic, clinical, and pathological features of extracranial AVMs.

## Materials and methods

### Patient selection

Formalin-fixed paraffin-embedded (FFPE) tissues obtained through resection from 30 patients with extracranial AVMs and 20 patients with lymphatic malformations (LMs) were retrieved from the pathology files of Osaka University Hospital. All vascular malformations were classified according to the classification system of the International Society for the Study of Vascular Anomalies [1]. The final diagnosis was confirmed by two pathologists (K.H. and Y.H.).

### Mutation analysis

Next-generation sequencing was performed using a custom panel, as previously described [21]. Genomic DNA was extracted from FFPE tissues showing AVMs and LMs using the QIAamp DNA FFPE Tissue Kit (Qiagen, Valencia, CA, USA). The gene panel was designed using SureDesign (https://earray.chem.agilent.com/suredesign. Accessed December 10, 2023) to cover all exons of genes associated with the RAS/RAF/MAPK and PI3K/AKT pathways (*KRAS*, *NRAS Proto-Oncogene, GTPase* (*NRAS*), *HRAS proto-oncogene*, *GTPase* (*HRAS*), *BRAF*, *MAP2K1*, *RAS p21 protein activator 1* (*RASA1*), *TEK*, *phosphatidylinositol-4, 5-bisphosphate 3-kinase catalytic subunit alpha* (*PIK3CA*), *AKT1*, and *phosphatase and tensin homolog* (*PTEN*)). Sequence libraries were prepared using the custom SureSelect Low-Input Target Enrichment System (Agilent Technologies, Inc. Santa Clara, CA, USA) and sequenced using Illumina MiSeq (Illumina, San Diego, CA, USA). Alissa Reporter ver1.3.3 (https://ap.reporter.alissa.agilent.com/. Accessed May 21, 2025) was used for variant calling. Intron DNA, non-coding DNA, and variant allele frequency < 1% were excluded. Variants obtained using panel sequencing were confirmed by Sanger sequencing with the primers (SI. 1).

### Histological and immunohistochemical analysis

Resected tissue samples were fixed with 10% formalin, routinely embedded in paraffin, cut into 4-μm thick serial sections, and used for hematoxylin–eosin, Elastica van Gieson, and immunohistochemical staining. Elastica van Gieson staining was used to determine whether the vessels in the AVM lesions were arteries or veins. Immunohistochemical staining was performed using Roche Ventana BenchMark GX Autostainer (Ventana Medical Systems, Tucson, AZ, USA). Primary antibodies against p44/42 MAPK (Erk1/2) (#4695; Cell Signaling Technology, Danvers, MA, USA), phospho-p44/42 MAPK (Erk1/2) (Thr202/Tyr204) (#4370; Cell Signaling Technology), and mitogen-activated protein kinase kinase kinase kinase 4 (MAP4K4) (HPA008476; Merk, Darmstadt, Germany) were used. Antibody for phospho-p44/42 MAPK (Erk1/2) detects endogenous levels of ERK1/ERK2 when dually phosphorylated at Threonine 202 and Tyrosine 204 of ERK1 (Threonine 185 and Tyrosine 187 of ERK2) and singly phosphorylated at Threonine 202 of ERK1. The expression levels of these proteins were assessed by two pathologists using a visual grading system based on the staining intensity, as previously described [21]. Undetectable, weak, moderate, and strong staining were defined as negative (score 0), low (score 1), intermediate (score 2), and high (score 3), respectively. H-scores were calculated using the following formula: H-score = 0 (% cells with score 0) + 1 (% cells with score 1) + 2 (% cells with score 2) + 3 (% cells with score 3).

### Spatial transcriptomics

Spatial transcriptomics and gene expression analysis were performed as previously described [21]. FFPE samples were cut into 5-μm thick sections. Visium libraries were prepared according to the Visium Spatial Gene Expression User Guide and sequenced using DNBSEQ-G400RS (MGI). The raw FASTQ files and histological images were processed using Space Ranger software (v2.1.1). The raw Visium files for each sample were read using Loupe Browser software (v8.1.1) to visualize the spatial expression using histological images. We obtained 175,243,789 and 148,386,748 sequence read counts and identified 37,654 and 47,352 median reads under tissue per spot in *MAP2K1*^*Q56P*^-mutant and *MAP2K1*^*K57N*^-mutant AVMs, respectively. We examined up-regulated genes at small vessel spots within each AVM compared to other spots or large vessel spots. Gene ontology enrichment analysis was performed using Metascape (https://metascape.org/. Accessed November 18, 2024).

### Statistical analyses

Data are expressed as means with standard deviation (±). Statistical analyses and graph creation were performed using Microsoft Excel and GraphPad Prism version 10 (La Jolla, CA, USA). Statistical significance was set at a p-value < 0.05. Data were tested using the Student’s t-test, Fisher’s exact test, Chi-square test of independence, and Tukey’s multiple comparison test.

## Results

### Mutational analyses in RAS/RAF/MAPK and PI3K/AKT pathways

**Fig. 1:**
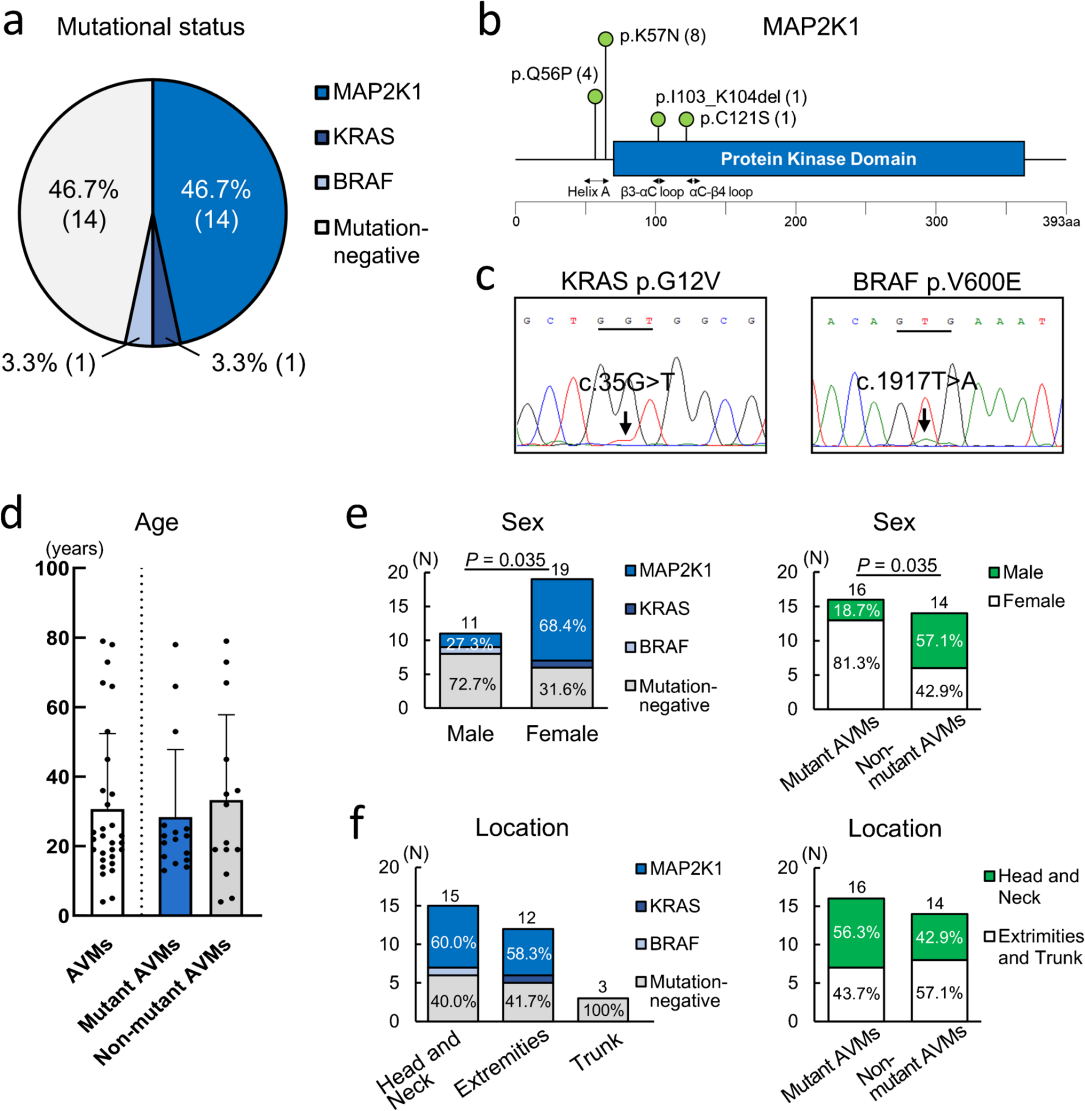
Molecular genetic analysis and association with clinical features in arteriovenous malformations. **a.** Pie chart showing the contribution of mutant genes in arteriovenous malformations (AVMs). **b.** The occurrence and distribution of various *MAP2K1* mutation sites. **c.** Direct gene sequencing showing chromatograms for *KRAS* p.G12V (c.35G > T) mutation and *BRAF* p.V600E (c.1917T > A) mutation. **d.** Age distribution in relation to mutation presence (white: all AVMs, blue: mutant AVMs, gray: non-mutant AVMs). The points indicate the ages of individual patients. **e.** Mutation presence distribution in relation to sex (left) and sex distribution in relation to mutation presence (right). **f.** Mutation presence distribution in relation to location (left) and location distribution in relation to mutation presence (right). P values were determined by Student’s T-test (for age), Fisher’s exact test (for sex), or Chi-square test of independence (for location)

Next-generation sequencing of the DNA of the 30 patients with extracranial AVMs detected genetic mutations in 16 (53.3%) patients (Fig. 1a). These include *MAP2K1* mutations in 14 (46.7%) patients and *KRAS* and *BRAF* mutations in one patient each (3.3%). All genetic mutations were somatic; no germline mutations were identified. Furthermore, these somatic mutations were mutually exclusive (Table 1). All identified mutations were detected in genes involved in the RAS/RAF/MAPK signaling pathway, and no mutations were detected in genes involved in the PI3K/AKT pathway. No mutations were detected in the remaining 14 of the 30 patients (46.7%) through our targeted sequencing analysis; thus, these patients were classified as having non-mutant AVMs. The most common variant in *MAP2K1* mutation was p.K57N (n = 8), followed by p.Q56P (n = 4), p.I103_K104del (n = 1) and p.C121S (n = 1) (Fig. 1b). Twelve of the 14 *MAP2K1* mutations were located in exon 2 (p.Q56 and p.K57) and the other two in exon 3 (p.I103 and p.C121) (Fig. 1b). The *KRAS* p.G12V mutation (exon 2) and *BRAF* p.V600E mutation (exon 15) have been termed hotspot mutations in various malignancies (Fig. 1c). The genetic characteristics of the present and previous large-scale studies are summarized in Table 1 and SI. 2.

### Clinical features

We evaluated 30 patients with AVMs (mean age: 30.67 years [range: 4–79 years, median: 21.5]; male-to-female ratio: 1:1.73 (11 male and 19 female) (Fig. 1d and 1e). AVMs occurred most frequently in the head and neck region (15 patients, 50.0%), followed by the extremities (12 patients, 40.0%) and trunk (3 patients, 10.0%) (Fig. 1f). All 30 patients had sporadic AVM (a single lesion in 29 patients and multiple lesions in one patient), with no personal or familial history of syndromes. Twenty-six patients had primary lesions, and four patients had recurrent lesions.

The mean ages of patients with mutant AVMs (*MAP2K1*-, *KRAS*-, and *BRAF*-mutant AVMs) and non-mutant AVMs were 28.38 (range: 13–78, median: 22.5) and 33.29 (range: 4–79, median: 26.5) years, respectively (Fig. 1d). Patients with mutant AVMs tended to be slightly younger than those without, but the difference was not statistically significant. The proportion of females was significantly higher in mutant AVMs (Fig. 1e). No difference was found in the location of AVM between mutant and non-mutant AVMs (Fig. 1f). The number of AVMs occurring in the trunk was small (Fig. 1f) and the analysis was performed in two groups: AVMs occurring in the head and neck region, and AVMs occurring elsewhere. Moreover, there was no difference in the number of lesions, whether the lesions were primary or recurrent, or the size of the resected lesion (mm^3^) between mutant and non-mutant AVMs. The genetic and clinical characteristics are summarized in SI. 3 and SI. 4.

### Histological features

**Fig. 2:**
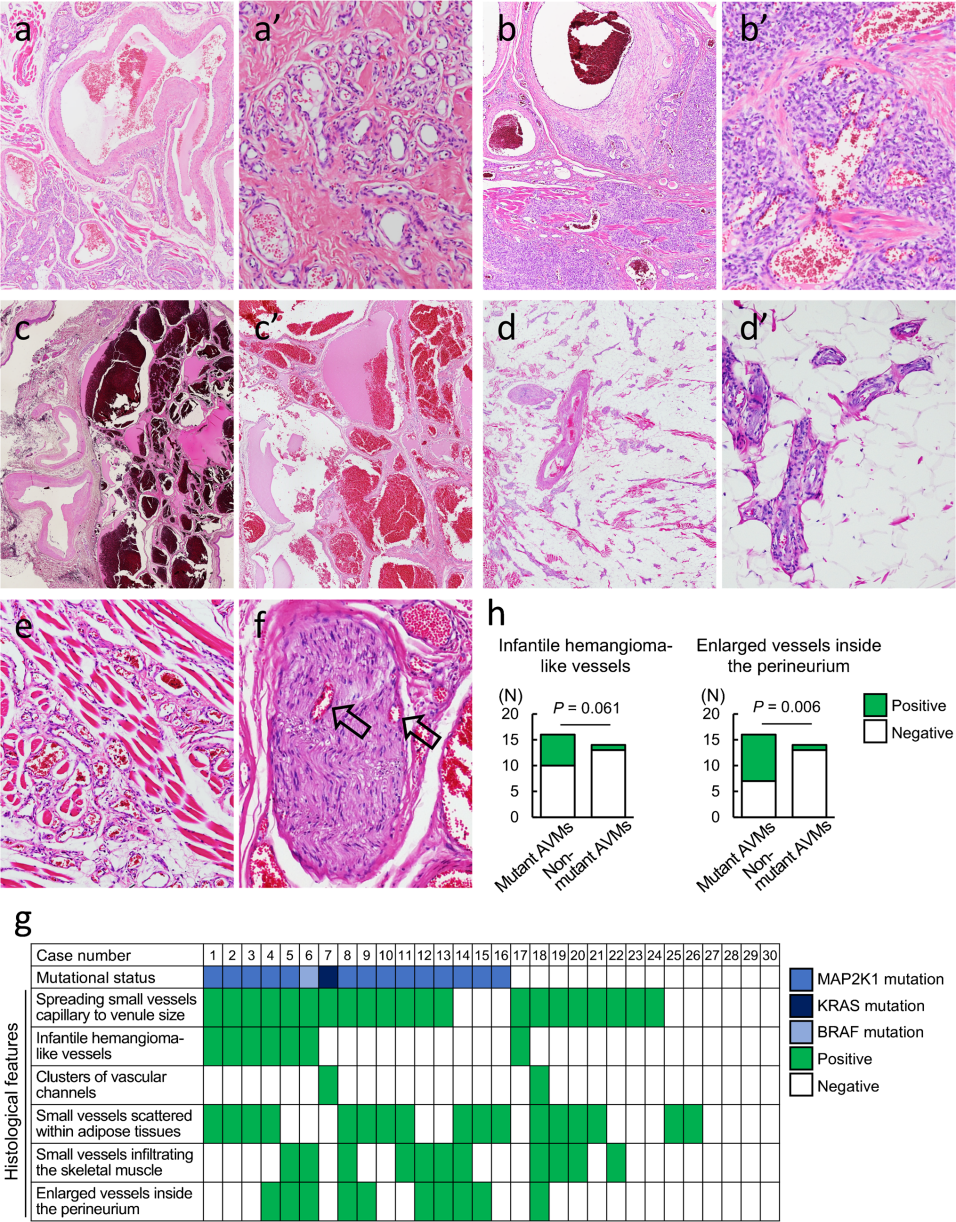
Association between histological features and mutational status in arteriovenous malformations. **a.** Arteriovenous malformations (AVMs) with spread of small vessels ranging from capillary size to venule size, often separated by fibrous tissues (a’: higher magnification of a). **b.** AVMs with infantile hemangioma-like small vessels (b’: higher magnification of b). **c.** AVMs, including clusters of venous channels with cavernous vascular spaces, predominate (c’: higher magnification of c). **d.** Small vessels scattered within adipose tissues (d’: higher magnification of d). **e.** Abnormal vessels infiltrating skeletal muscle fibers. **f.** Enlarged small vessels inside the perineurium (arrows: enlarged vessels). **g.** Chart showing individual characteristics of mutational status and histological features. **h**. Distribution of histological features in relation to mutation presence (left: infantile hemangioma-like small vessel, right: enlarged small vessels inside the perineurium). P values were determined by the Chi-square test of independence

Histologically, AVMs vary in appearance, and their common feature is disproportionately large vessels, including arteries and veins. The arteries showed focal dissolution of the internal elastic lamina and neointimal cushion. Enlarged veins showed thickened walls with an artery-like appearance, and some veins may have thinned vascular walls. An overall increase in small vessels that resembled arterioles, venules, and capillaries was observed, and these were considered to have formed abnormal networks (Fig. 2a-2c’).

The abnormal small vessels ranging from capillary size to venule (or arteriole) size were widely distributed (Fig. 2a), showed wide lumens, and were often separated by fibrous tissues. Further, they occasionally showed small foci or lobular growth, similar to the infantile hemangiomas (Fig. 2b). In these cases, plump endothelial cells and perivascular cells formed small back-to-back capillaries with inconspicuous lumens (Fig. 2b’). Clusters of venous malformation-like vascular channels were rarely more prominent than small vessels (Fig. 2c). Additionally, abnormal vessels scattered within adipose tissues (Fig. 2d), infiltrating skeletal muscle fibers (Fig. 2e), and enlarged small vessels inside the perineurium (Fig. 2f) were occasionally observed. These abnormal vessels often showed multiple histological patterns in the same case (Fig. 2g).

Mutant AVMs were more likely to have an infantile hemangioma-like vessel pattern (6/16, 37.5%) than non-mutant AVMs (1/14, 7.1%) (Fig. 2h). Mutant AVMs had more enlarged vessels inside the perineurium (9/16, 56.3%) than non-mutant AVMs (1/14, 7.1%) (Fig. 2h). No significant differences were observed in other histological features between mutant and non-mutant AVMs (Fig. 2g).

### RAS/RAF/MAPK pathway activation

**Fig. 3:**
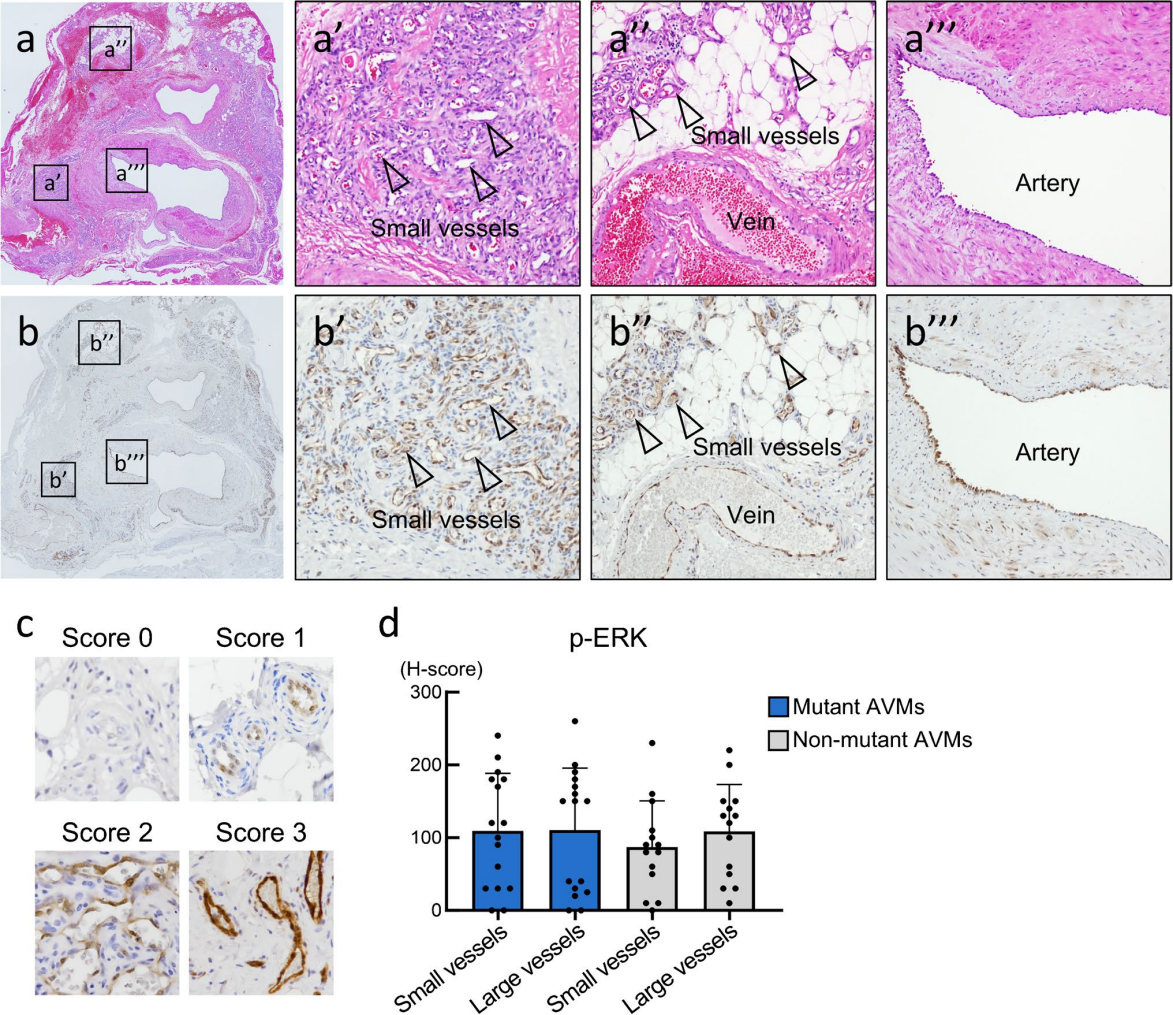
Immunohistochemical analyses of the expression of MAPK pathway in arteriovenous malformations. **a, b.** Representative arteriovenous malformations (AVMs) with hematoxylin–eosin staining (a) and immunohistochemical staining for phosphorylated extracellular signal-regulated kinase (p-ERK) (b). a’, a’’, and a’’’ show higher magnifications of a, and immunohistochemical staining of the same corresponding areas are shown as b’, b’’, and b’’’ (arrowheads: small vessels). **c.** Representative immunohistochemical staining pattern of p-ERK. **d.** Distribution of p-ERK expression in relation to the presence of mutations. The points indicate the H-scores of individual patients. P values were determined by Tukey’s multiple comparison test

We analyzed the expression patterns of ERK, a downstream effector of *MAP2K1*. Non-phosphorylated ERK1/ERK2 (total ERK) was diffusely detected in abnormal vessels in AVMs, as well as in other tissues, including ECs of normal vessels, fibroblasts, and peripheral nerves (SI. 5a). Phosphorylated ERK1/ERK2 (p-ERK) was expressed specially in the ECs of abnormal vessels in AVMs (Fig. 3a and 3b). p-ERK expression was observed in the ECs of small vessels (Fig. 3a’, 3a’’, 3b’, and 3b’’) as well as in large vessels (arteries and veins); however, its expression in large vessels was occasionally weak (Fig. 3a’’, 3a’’’, 3b’’ and 3b’’’). The expression levels of p-ERK were not significantly different among small vessels with mutant AVM, large vessels with mutant AVMs, small vessels with non-mutant AVMs, or large vessels with non-mutant AVMs (Fig. 3c and 3 d). In contrast, p-ERK was either undetectable or very weak in normal vessels (SI. 5b).

Next, we examined the ERK expression patterns in LMs associated with genetic mutations unrelated to the MAPK pathway. Mutational analyses of LMs revealed that only somatic *PIK3CA* mutations involved in the PI3K/AKT pathway were detected, and no mutations were detected in genes involved in the RAS/RAF/MAPK pathway (SI. 5c). Total ERK expression levels did not show notable differences between AVMs and LMs (SI. 5a). p-ERK was weak in most lymphatic vessels in LMs, and p-ERK expression levels in both mutant and non-mutant AVMs were significantly higher than those in LMs (SI. 5b and 5 d).

### Transcriptomic profiles

**Fig. 4:**
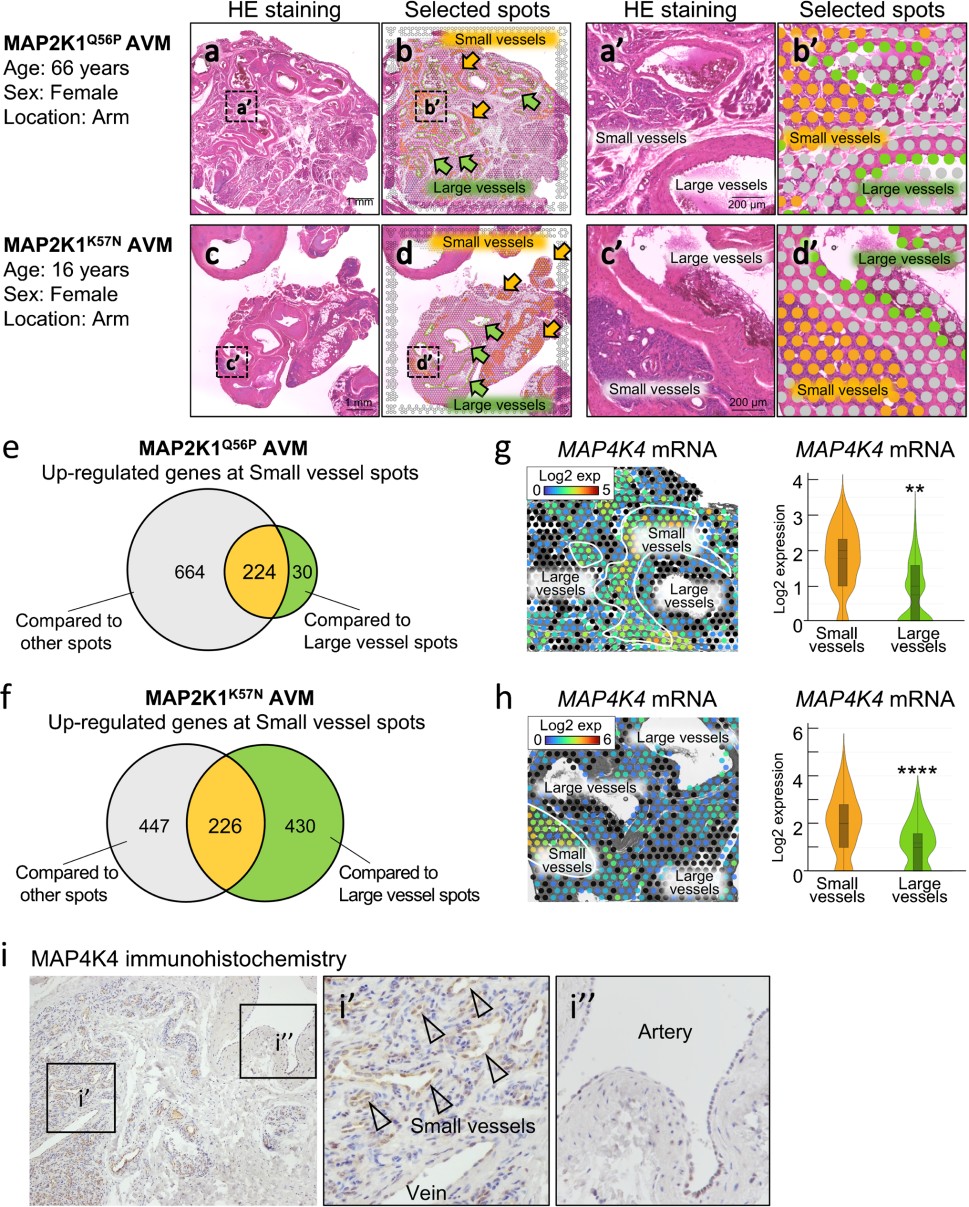
Spatial transcriptomics in arteriovenous malformations. **a-d.** Hematoxylin–eosin (HE) staining (a and c) and selected spots (b and d) in a *MAP2K1*^*Q56P*^-mutant arteriovenous malformation (AVM) (a and b) and a *MAP2K1*^*K57N*^-mutant AVM (c and d). a', b', c', and d'were higher magnifications of a, b, c, and d, respectively. “Small vessel” (orange arrows and circles) and “Large vessel” (green arrows and circles) indicate selected spots of endothelial cells (ECs) of small vessels and large vessels (including arteries and veins), respectively. **e, f.** Up-regulated genes at small vessel spots compared with the other spots or large vessel spots in a *MAP2K1*^*Q56P*^-mutant AVM (e) and a *MAP2K1*^*K57N*^-mutant AVM (f). **g, h.** Expression of *mitogen-activated protein kinase kinase kinase kinase 4* (*MAP4K4*) mRNA in spatial transcriptomic images and violin plots in a *MAP2K1*^*Q56P*^-mutant AVM (g) and a *MAP2K1*^*K57N*^-mutant AVM (h), respectively. **i.** Representative immunohistochemical staining of MAP4K4 in *MAP2K1*-mutant AVMs. i’ shows the region of small vessels, and i’’ shows the region of the artery (i’ and i’’: higher magnifications of i, arrowheads: small vessels). P-values were determined by Benjamini–Hochberg correction. P** < 0.01, P**** < 0.0001

Spatial transcriptomics was performed for *MAP2K1*^*Q56P*^- and *MAP2K1*^*K57N*^-mutant AVMs (Fig. 4a-4d). They showed similar findings regarding sex, location, and small vessel type (Fig. 4a and 4c). *CD31* (markers for ECs) and *CDH5* (markers for ECs of blood vessels) mRNA were highly expressed in both “small vessel spots” and “large vessel spots,” with no significant difference between their spots (SI. 6a). We identified 224 and 226 genes that were highly expressed in small vessel spots in *MAP2K1*^*Q56P*^- and *MAP2K1*^*K57N*^-mutant AVM, respectively (Fig. 4e, 4f, SI. 7 and SI. 8). Gene ontology analysis was performed to investigate the functions of the 100 genes whose expression increased in small vessel spots common to *MAP2K1*^*Q56P*^- and *MAP2K1*^*K57N*^-mutant AVM (SI. 9). The findings revealed that the genes were mainly involved in “positive regulation of cell adhesion,” “positive regulation of cell migration,” “negative regulation of locomotion, and “blood vessel development” (SI. 6b and SI. 10). Within the biological process, we focused on the category “MAPK cascade” (SI. 6b). This category contained nine genes as follows: *MAP4K4*, *ZFP36L1*, *IGFBP3*, *SH2D3C*, *SPRY1*, *ITPKB*, *PLVAP*, *CD36*, and *BOC* (Fig. 4g, 4 h, and SI. 6c). In both *MAP2K1*^*Q56P*^- and *MAP2K1*^*K57N*^-mutant AVMs, *MAP4K4* mRNA was highly expressed in the small vessel spots compared with the large vessel spots (Fig. 4g and 4 h). MAP4K4 was expressed almost exclusively in the ECs of small vessels in *MAP2K1*-mutant AVMs (Fig. 4i and 4i’). MAP4K4 was either negative or very weakly expressed in the ECs of large vessels (Fig. 4i and 4i’’).

## Discussion

This is the first study to report comprehensive clinical, histological, immunohistochemical, and genetic analyses of extracranial AVMs. The present study reported some characteristic clinicopathological differences associated with genetic mutations. Patients with mutant AVMs were slightly younger than those with non-mutant AVMs, but the difference was not statistically significant (Fig. 1d). Mutations were detected more frequently in females than in males (Fig. 1e). Histologically, differences existed in the small vessel types between mutant and non-mutant AVMs (Fig. 2). Mutant AVMs showed more infantile hemangioma-like small vessels and more enlarged small vessels inside the perineurium, unlike non-mutant AVMs, which rarely showed these findings (Fig. 2).

We evaluated 94 patients with extracranial AVMs in the present study and previous genetic studies (SI. 4) [5, 14–16]. Patients with mutant AVMs (65 patients, mean: 23.43 years, median: 21 years, range: 1–79 years) were slightly younger than those with non-mutant AVMs (29 patients, mean: 31.48 years, median: 23 years, range: 4–78 years) (*p* = 0.036). Considering the results of the present and previous studies, extracranial AVMs with genetic mutations may be more common in young females. A previous study on extracranial AVMs reports that *KRAS*-mutant AVMs are more clinically aggressive and have a higher recurrence rate than *MAP2K1*-mutant AVMs [5]. Both mutations in *KRAS* and *MAP2K1* affect the same pathway; however, *KRAS* is located upstream of *MAP2K1* in the signaling cascade. Sissy et al. suggested that mutations in *KRAS* affect pathways other than the RAS/MAPK signaling cascade, leading to a more severe phenotype than mutations in *MAP2K1* [5]. A similar phenotype-genotype correlation has been reported in venous malformations (VMs) involved in mutations of *TEK* and *PIK3CA*. *TEK* is located upstream of *PIK3CA* in the PI3K/AKT pathway, and *TEK*-mutant VMs occur more often in younger patients and show histologically more frequent skin involvement [21]. Significant genotype–phenotype correlations in the clinicopathological features could suggest and support the presence of specific mutations in extracranial AVMs. In the present study, only one *KRAS*-mutant case and one *BRAF*-mutant case occurred (Fig. 1a). Further accumulation of extracranial AVMs is needed to investigate the differences in clinicopathological findings among AVM-mutant genotypes.

*MAP2K1* mutation alleles are only present in ECs extracted from AVM lesions, suggesting that ECs affect extracranial AVM progression [14, 19]. To the best of our knowledge, this is the first study to report the transcriptomics of ECs in extracranial AVM lesions in vivo. AVMs typically comprise arteries, veins, and abnormal small-vessel networks. Spatial transcriptomics enables detailed examinations of gene expression in each vascular type. Our transcriptomic findings revealed that the ECs of small vessels (small vessel spots) showed up-regulation of genes involved in the positive regulation of cell adhesion, positive regulation of cell migration, and blood vessel development (SI. 6b and SI. 10). These results are consistent with previous studies showing that *MAP2K1*-mutant-transduced ECs enhance migratory behavior and angiogenesis [15, 19, 22]. Many candidate genes involved in extracranial AVM pathogenesis have been identified (SI. 6b and SI. 10). Biological process of Gene Ontology termed “MAPK cascade” includes nine genes, but the involvement of these factors in extracranial AVM pathogenesis has not been reported (Fig. 4g, 4 h, and SI. 6c). MAP4K4 was listed in many other biological process terms besides “MAPK cascade,” such as “positive regulation of cell adhesion,” “positive regulation of cell migration,” and “positive regulation of phosphorus metabolic process.” MAP4K4 regulates several biological processes, including angiogenesis, embryonic development, metabolism, inflammation, cardiovascular disease, and cancer [23–26]. MAP4K4 in ECs is essential for vascular development and controls cell migration [23]. Loss of MAP4K4 in ECs decreases membrane dynamics, slows cell migration, and impairs angiogenesis [23]. MAP4K4 inhibitors show promise in treating pathological angiogenesis in mouse models of cancer and eye diseases [23, 27]. The present study revealed that MAP4K4 was highly expressed in ECs, especially in the small vessels of extracranial AVMs (Fig. 4i). Further studies are needed to determine whether MAP4K4 is involved in AVM progression and to identify MAP4K4 as a potential therapeutic target for extracranial AVMs. Analysis of the genes identified by in vivo transcriptomics may lead to a more detailed understanding of the mechanisms underlying AVM progression. Downstream pathway analysis of RAS/RAF/MAPK mutations in AVMs may further identify targeted medicines in the future

The present study identified somatic mutations in the RAS/RAF/MAPK pathway and revealed a high activation of the downstream effector ERK in extracranial AVMs. Combining the data from the present study with previous genetic studies on extracranial AVMs, the prevalence of *MAP2K1*, *KRAS*, *BRAF* mutations, and *RASA1* mutations was 40.6% (41/101), 9.9% (10/101), 4.0% (4/101), and 2.0 (2/101), respectively (Table 1) [5, 14, 15]. These somatic mutations were mutually exclusive. The variants of *MAP2K1*, *KRAS*, or *BRAF* mutations identified in the present study have also been identified in malignant tumors, where they play a ‘driver’ role in tumor growth and for which targeted therapies have been developed and are widely used [10–12]. Given the similarity of the targets, treatment with MEK (encoded by *MAP2K1* gene) inhibitors and other agents in the RAS/RAF/MAPK pathway could offer a new therapeutic strategy for clinicians managing patients with AVMs [9, 13]. Some patients with severe extracranial AVMs were treated off-label with trametinib (MEK inhibitor) and showed favorable responses, including reduction in lesion volume and associated symptoms [13, 28, 29]. Moreover, prospective phase II trials using trametinib included difficult-to-treat AVMs (EudraCT number: 2019–003573-26) and extracranial AVMs (NCT04258046). To our knowledge, the present study is the first to report the expression of p-ERK, indicating the activation of the RAS/RAF/MAPK pathway, in *in vivo* extracranial AVMs (Fig. 3d). We demonstrated that p-ERK was highly expressed in the ECs of AVMs, regardless of mutational status or vessel type, compared to that in normal vessels (Fig. 3d). Considering that total ERK was diffusely detected in various types of cells, post-translational modifications of ERK are specifically advanced in the ECs of extracranial AVMs. Moreover, p-ERK was highly expressed in AVMs compared to LMs involved in gene mutations in the PI3K/AKT pathway (SI. 5 d) [30]. These results suggest that the RAS/RAF/MAPK pathway may be activated in extracranial AVMs through the mutations in genes of the RAS/RAF/MAPK pathway or in genes associated with ERK activation, outside of the PI3K/AKT pathway. These results may provide therapeutic evidence for targeting the RAS/RAF/MAPK pathway, including the use of MEK inhibitors, in the treatment of AVM. Further studies are needed to determine the possibility of predicting the response to MEK inhibitor treatment or to evaluate the feasibility of p-ERK expression levels as a biomarker of treatment response.

Extracranial AVMs are mainly associated with *MAP2K1* mutations, while brain AVMs are linked to *KRAS* mutations. Both share genetic mutations within the same pathway [17, 18], suggesting that the two conditions may share a similar underlying mechanism [17, 18]. Concerning brain AVM research, Wälchli et al. (2024) reported a single-cell atlas of brain AVMs through single-cell RNA sequencing analysis of ECs derived from patients with brain AVMs [31]. However, distinguishing ECs from abnormal small-vessel networks versus ECs in arteries, veins, and normal vessels remains challenging. Our spatial transcriptomics data, which identifies small-vessel specific genes, offers a more precise understanding of the mechanisms underlying brain AVM pathogenesis and highlights their potential as biomarkers for diagnosis or therapeutic targeting.

The present study had some limitations. First, treatment history, such as embolization or administration of medications before the resection of AVM lesions, was not considered. This is attributed to the complex treatment strategies that differ depending on the age, disease location, and clinical symptoms of the patients. Second, the mutational status of approximately 40–50% of extracranial AVMs remains unknown. These AVMs are likely caused by infrequent mutations in several genes associated with promoting cell proliferation and growth of AVMs, as suggested by Sissy et al. [5] Further genetic studies on these patients would provide critical support for the current genetic theory of AVMs pathogenesis.

## Conclusions

Significant genotype–phenotype correlations in the clinical and pathological features of extracranial AVMs were observed among individuals with genetic mutations, indicating gene-specific effects. These features suggest the presence of specific mutations in AVMs. The detailed analysis of gene-specific effects, focusing especially on abnormal small-vessel networks, offers insights into the underlying mechanisms of AVM pathogenesis and provides an opportunity to identify selected targeted therapies in the future.

## Supplementary Information

Below is the link to the electronic supplementary material.

Supplementary file1 (PDF 2531 KB)

Supplementary file2 (XLSX 222 KB)

Supplementary file3 (XLSX 296 KB)

Supplementary file4 (XLSX 11 KB)

Supplementary file5 (XLSX 108 KB)

## Data Availability

Data supporting the findings of this study are available upon request from the corresponding authors.
