# Supplementary material for: Pathogenic mechanism of extracranial arteriovenous malformations: insights from clinical, pathological, and genetic analyses
Source: Virchows Arch. 2025 Jul 2;488(5):1137–48. doi: 10.1007/s00428-025-04158-7 (PMC13175998; doi:10.1007/s00428-025-04158-7)
Supplement: Supplementary file 1 — Supplementary file1 (PDF 2531 KB) [file 428_2025_4158_MOESM1_ESM.pdf]

Pathogenic Mechanism of Extracranial Arteriovenous Malformations: Insights from Clinical, Pathological, and Genetic Analyses

Virchows Archiv European Journal of Pathology

Katsutoshi Hirose[1], Yumiko Hori[2,3,*], Kazuaki Maruyama[4], Daisuke Motooka[5], Kenji Hata[6], Shinichiro Tahara[2], Takahiro Matsui[2], Satoshi Nojima[2], Masaharu Kohara[2], Kyoko Imanaka-Yoshida[4,7], Satoru Toyosawa[1] and Eiichi Morii[2.]

[1]Department of Oral and Maxillofacial Pathology, Osaka University Graduate School of Dentistry, 1-8 Yamadaoka, Suita, Osaka 565-0871, Japan; ORCID; 0000-0003-4148-1106.

[2]Department of Pathology, Osaka University Graduate School of Medicine, 2-2 Yamadaoka, Suita, Osaka 565-0871, Japan.

[3]Department of Central Laboratory and Surgical Pathology, NHO Osaka National Hospital, 2-1-14 Hoenzaka, Chuo-ku, Osaka 540-0006, Japan.

[4]Department of Pathology and Matrix Biology, Mie University Graduate School of Medicine, 2-174 Edobashi, Tsu, Mie 514-8507, Japan.

[5]Genome Information Research Center, Research Institute for Microbial Diseases, Osaka University, 3-1 Yamadaoka, Suita, Osaka 565-0871, Japan.

[6]Department of Molecular and Cellular Biochemistry, Osaka University Graduate School of Dentistry, 1-8 Yamadaoka, Suita, Osaka 565-0871, Japan.

[7]Mie University Onco-Cardiology Research Center, 2-174 Edobashi, Tsu, Mie 514-8507, Japan.

***Corresponding author:**

Yomiko Hori (yumiko-hori@molpath.med.osaka-u.ac.jp)

Department of Pathology, Osaka University Graduate School of Medicine, 2-2 Yamadaoka, Suita, Osaka 565-0871, Japan.

Tel: +81-6-6879-3711

Department of Central Laboratory and Surgical Pathology, NHO Osaka National Hospital, 2-1-14 Hoenzaka, Chuo-ku, Osaka 540-0006, Japan.

Tel: +81-6-6942-1331

Supplementary Information 1.

Primer sequences used in the present study

Gene	Exon	Forward primer	Reverse primer
MAP2K1	Exon 2	TGACTTGTGCTCCCCACTTT	GTCCCCAGGCTTCTAAGTACC
MAP2K1	Exon 3	TCATCCCTTCCTCCCTCTTT	CTCTTAAGGCCATTGCTCCA
KRAS	Exon 2	GTGTGACATGTTCTAATATAGTCA	GAATGGTCCTGCACCAGTAA
BRAF	Exon 15	TAAACTCTTCATAATGCTTGCTCTGA<br>T	AACTCAGCAGCATCTCAGGGCCA<br>A

MAP2K1, mitogen-activated protein kinase kinase 1; KRAS, KRAS proto-oncogene, GTPase; BRAF, B-Raf proto-oncogene, serine/threonine kinase.

Supplementary Information 2.

Summary of distribution of variants of somatic mutations identified in the present study and other major genetic studies of extracranial arteriovenous malformations

Gene (cases)	Variants	Number of cases
MAP2K1 (48)[5,14-16]	p.F53_Q58delinsL	1
	p.F53L+p.D67Y	1
	p.Q56P	10
	p.K57N	30
	p.Q58_E62del	3
	p.I103_K104del	1
	p.C121S	1
	p.C121S+p.P124L	1
KRAS (10)[5,15]	p.G12C	1
	p.G12D	2
	p.G12V	2
	p.Q61H	5
BRAF (5)[5,15,16]	p.V600E	5
RASA1 (2)[5]	p.R789*	1
	Deletion exon 5	1

MAP2K1, mitogen-activated protein kinase kinase 1; KRAS, KRAS proto-oncogene, GTPase; BRAF, B-Raf proto-oncogene, serine/threonine kinase; RASA1, RAS p21 protein activator 1.

Supplementary Information 3.

Summary of genetic and clinical characteristics in the present study of extracranial arteriovenous malformations

Case	Mutational gene	Age (y) /Sex	Location	Number of lesions (single/multiple/several organs)	Primary or recurrent lesion	Size of resected lesion (mm)	Other complicated lesions
1	MAP2K1	15/F	Extremities	Single	Primary	7x8x70	None
2	MAP2K1	24/F	Extremities	Single	Primary	14x16x20	None
3	MAP2K1	17/F	Extremities	Single	Primary	12x15x28	Ulceration
4	MAP2K1	14/F	Extremities	Single	Primary	5x7x70	None
5	MAP2K1	66/F	Extremities	Single	Primary	10x25x33	Ulceration
6	BRAF	21/M	Head & Neck	Single	Primary	17x17x27	None
7	KRAS	78/F	Extremities	Multiple	Primary	10x15x15	Tingling in fingers,
8	MAP2K1	23/F	Head & Neck	Single	Primary	12x16x48	None
9	MAP2K1	16/F	Head & Neck	Single	Primary	10x11x26	Swelling
10	MAP2K1	23/M	Extremities	Single	Primary	12x17x27	Necrosis, Ulceration
11	MAP2K1	22/F	Head & Neck	Single	Recurrent	9x12x19	None
12	MAP2K1	53/F	Head & Neck	Single	Primary	42x60x100	Ulceration, Bleeding
13	MAP2K1	25/F	Head & Neck	Single	Primary	11x14x75	Swelling
14	MAP2K1	26/F	Head & Neck	Single	Primary	11x13x22	None
15	MAP2K1	18/M	Head & Neck	Single	Primary	15x18x22	None
16	MAP2K1	13/F	Head & Neck	Single	Primary	4x7x24	Swelling
17	Negative	5/M	Head & Neck	Single	Primary	11x15x17	Swelling
18	Negative	35/F	Trunk	Single	Primary	18x35x85	None
19	Negative	12/M	Extremities	Single	Primary	25x32x62	None
20	Negative	21/M	Head & Neck	Single	Primary	14x38x45	None
21	Negative	36/M	Trunk	Single	Primary	90x200x270	Swelling
22	Negative	19/M	Head & Neck	Single	Primary	17x17x42	None
23	Negative	19/M	Extremities	Single	Primary	25x43x52	None
24	Negative	19/F	Head & Neck	Single	Primary	10x21x23	None
25	Negative	4/M	Head & Neck	Single	Primary	15x33x68	None
26	Negative	79/F	Trunk	Single	Primary	43x207x293	Ulceration, Bleeding
27	Negative	67/F	Extremities	Single	Recurrent	6x12x13	Swelling, Pain
28	Negative	73/F	Extremities	Single	Recurrent	8x12x12	None
29	Negative	32/F	Head & Neck	Single	Recurrent	16x27x45	None
30	Negative	45/M	Extremities	Single	Primary	15x17x69	Necrosis

MAP2K1, mitogen-activated protein kinase kinase 1; BRAF, B-Raf proto-oncogene, serine/threonine kinase; KRAS, KRAS proto-oncogene, GTPase.

Supplementary Information 4.

Age distribution in relation to mutation presence in the present study and other major genetic studies of extracranial arteriovenous malformations

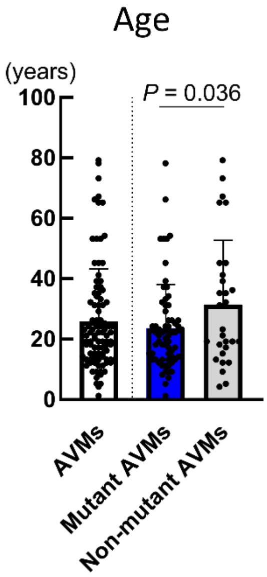

Age distribution in relation to mutation presence in extracranial arteriovenous malformations (AVMs) (white: all AVMs, blue: mutant AVMs, gray: non-mutant AVMs). The points indicate the ages of individual patients. P values were determined by Student's T test.

Supplementary Information 5.

Immunohistochemical analyses of the expression of MAPK pathway in normal tissues and vascular malformations

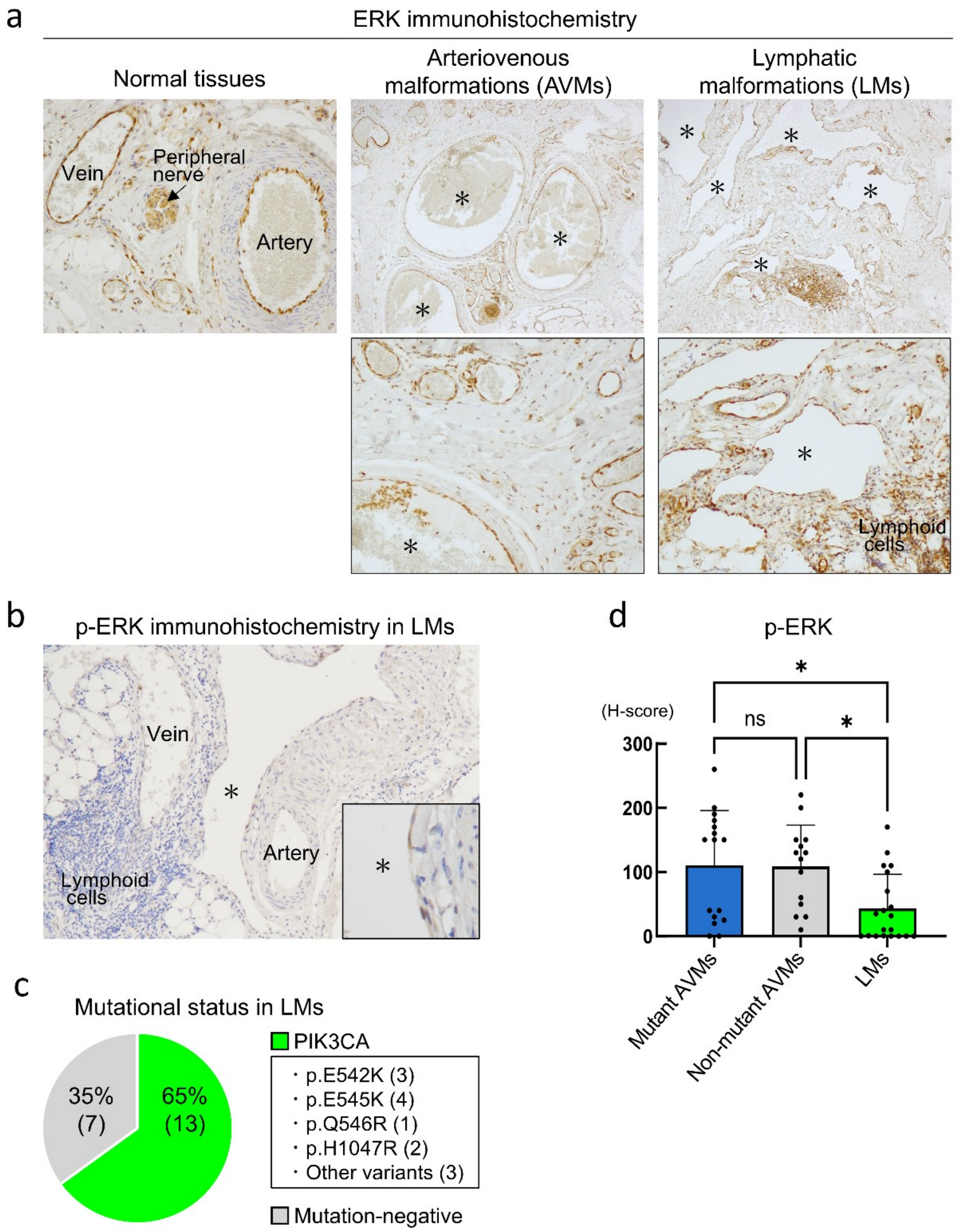

a. Representative normal tissues, arteriovenous malformations (AVMs), and lymphatic malformations (LMs) with immunohistochemical staining for non-phosphorylated extracellular signal-regulated kinase (total ERK) (asterisks: lumen of malformed vessels, black boxes: higher magnifications). **b.**

Representative LMs with immunohistochemical staining for phosphorylated extracellular signal-regulated kinase (p-ERK) (asterisk: lumen of malformed lymphatic vessels, inset: higher magnification). **c.** Pie chart showing the contribution of mutant genes in LMs. PIK3CA, phosphatidylinositol-4, 5-bisphosphate 3-kinase catalytic subunit alpha. **d.** Distribution of p-ERK expression in mutant AVMs, non-mutant AVMs, and LMs. Points indicate the H-scores of individual patients. P values were determined by Tukey's multiple comparison test. P*<0.05.

Supplementary Information 6. mRNA expression of genes related in blood vessel and "MAPK cascade" in arteriovenous malformations

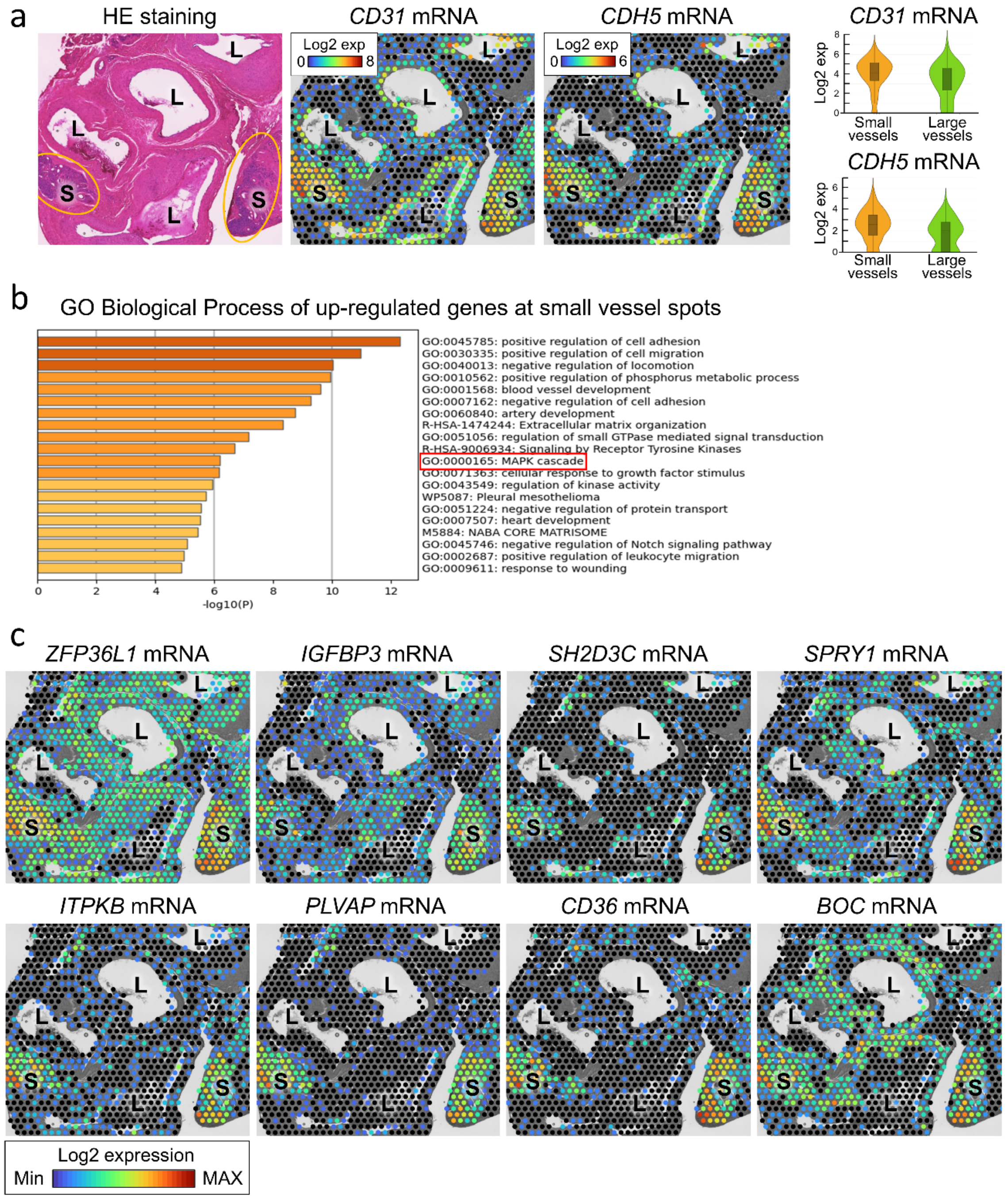

a. Hematoxylin-eosin (HE) staining, and the expression levels of *CD31* mRNA (maker for ECs) and *CDH5* mRNA (marker for ECs of blood vessels) in spatial transcriptomic images and violin plots of two types of vessels (S; small vessels, L; large vessels). P-values were determined by Benjamini-Hochberg correction. **b**. Gene ontology (GO) analysis of the up-regulated genes at small vessel spots

common to a *MAP2K1*^{Q56P}-mutant arteriovenous malformation (AVM) and a *MAP2K1*^{K57N}-mutant AVM. **c.** Expression levels of nine genes related to the MAPK cascade (excluding *MAP4K4*) in spatial transcriptomic images of a *MAP2K1*^{N57N}-mutant AVM. S, small vessels; L, large vessels; ZFP36L1, ZFP36 ring finger protein like 1; IGFBP3, insulin-like growth factor binding protein 3; SH2D3C, SH2 domain containing 3C; SPRY1, sprouty RTK signaling antagonist 1; ITPKB, inositol-trisphosphate 3-kinase B; PLVAP, plasmalemma vesicle-associated protein; CD36, CD36 molecule; BOC, BOC cell adhesion-associated oncogene regulated.
